# Supplementary material for: Pattern of Urban Flora in Intra-City Railway Habitats (Alexandria, Egypt): A Conservation Perspective
Source: Biology (Basel). 2021 Jul 22;10(8):698. doi: 10.3390/biology10080698 (PMC8389201; doi:10.3390/biology10080698)
Supplement: Supplementary file 1 [file biology-10-00698-s001.zip › biology-1301942-supplementary.pdf]

biology

Table S1. Properties of sampled stands.

Stand No.	Location	Stand size (m^2)		Coordinates		Alt. (m)	Soil Description	Human Impact	Type of Urbanization	Total Cover (%)	First Dominant Species	Population Density/ km² in 2019
		W	L	Long.	Lat.							
1	Alexandria Main Station (a)	5	15	29° 54.383-	31° 11.533-	40	Loamy Sandy Compact	Traffic pollution, Oil, Railway line	Settlements	2	*Sisymbrium irio, Urospermum picroides*	21,376
2	Alexandria Main Station (b)	30	200	29° 54.383-	31° 11.650-	33	Loamy Sandy Compact	Infra structure	Settlements	20	*Cynodon dactylon*	21,376
3	Alexandria Main Station (c)	30	200	29° 54.383-	31° 11.650-	33	Loamy Sandy some parts clay sand but compact	Infrastructure, solid waste	Settlements	30-35	*Avena fatua*	21,376
4	Alexandria-Matrouh railway	50	300	29° 55.117-	31° 10.950-	14	Compact Clay Loamy	Traffic pollution, Oil, Railway line, Solid waste, Grazing	Settlements	≥78	*Pharagmitis, Avena fatua*	22,663
5	El-Hadra-Wabour EL-Mia	50	200	29° 55.504-	31° 12.005-	17	Compact Soil with small gravel (Mixed Sand)	Solid waste, Sewage, Demolished parts, Air Pollution and Traffic Pollution	Natural	≥78	*Emex spinosa, Chenopodium murale*	42,289
6	Tarik El-Hadra (a)	60	100	29° 55.598-	31° 12.064-	11	Compact Soil with small gravel (Mixed Sand)	Solid waste, Sewage, Demolished parts	Natural Train Maintenance Station	≥78	*Emex spinosa, Avena fatua*	42,289
7	Tarik El-Hadra (b)	50	100	29° 55.623-	31° 12.083-	14	Compact Soil with small gravel (Mixed Sand)	Solid waste, Sewage, Demolished parts	Natural	≥78	*Emex spinosa, Chenopodium murale*	42,289
8	El-Hadra Station	150	200	29° 55.732-	31° 12.175-	17	Compact (Clay Loamy)	Solid waste, Fire, Refuse	Natural	50-60	*Avena fatua Mesymbrianthumun crystallinum*	42,289
9	El-Hadra-Behind Prison	40	200	29° 56.402-	31° 13.014-	9	Compact (Clay Loamy) with gravel	Solid waste, infrastructure	Natural, urbanization	40-50	*Cynodon dactylon Avena fatua*	42,289
10	El-Ibrahemia	80	100	29° 56.142-	31° 12.684-	35	Compact with gravel (loamy sand)	Solid waste, Settlements	Urbanization, Solid waste, natural	≥78	*Cynanchum acutum Veronica anagalis-aquatica*	42,289
11	Sidi Gaber Station	40	70	29° 56.402-	31° 13.044-	30	Compact Soli (Clay), with boulders of rockes	Urbanization (Settlements, Solid waste, Domestic waste)	Urbanization, Natural	20-30	*Chenopodium alba, Chenopodium murale*	15,025
12	Sidi Gaber (Kafr Abdou)	100	300	29° 57.139	31° 13.271	1	Calcareous with course stone (gravel & Boulders) (Mixed Sand)	Fire, waste places	Urbanization, Natural	≥78	*Emex spinose, Chenopodium murale*	15,025

Stand No.	Location	Stand size (m^2)		Coordinates		Alt. (m)	Soil Description	Human Impact	Type of Urbanization	Total Cover (%)	First Dominant Species	Population Density/km² in 2019
		W	L	Long.	Lat.							
13	Sidi Gaber-El-Nehas	100	500	29° 57.084	31° 13.269	1	Silt, Coarse Stone (mixed Sand)	Land Waste, Air Pollution from Motor Traffic, Settlement, Near industrial area	Urbanization, Natural	≥78	*Avena fatua, Emex spinosa*	15,025
14	Raml-ElMery	30	40	29° 58.587	31° 17.342	19	Calcareous Coarse Particles-Dumbing sites with Solid Waste	Overpopulation, highly impacted, Settlement, Animal Feces, animal Shelters, high traffic	Urbanization, Natural	35	*Sisymbrium irio, Malva parviflora*	20,105
15	Vectoria-Noqrashy	30	40	29° 58.755	31° 14.086	1	Gravel Clay	Overpopulation, Heavy Traffic, Solid waste, Local Market	Urbanization, Industrial	25-50	*Avena fatua, Deplotaxis harra*	20,105
16	Derbala	60	60	29° 59.115	31° 15.097	9	Loamy silty with gravel	Overpopulation, Heavy Traffic, Solid waste, Local Market, Waste from Vegetable Market, Fire	Urbanization, Natural	25-50	*Sismbarym, Avena, Deplotaxis, Malva*	20,105
17	Asafra-45 Street	60	60	30° 0.077	31° 15.938	1	Gravel Clay	Solid Waste, Overpopulation, Cabbage Site	Organic Solid Waste,		*Avenna, Urospermum, Deplotaxis, Centaurrea*	26,052
18	El-Eslah	30	50	30° 2.567	31° 17.148	-3.5	Clay, Loam	Solid waste	Roadside ,	50-75	*Conyza, Disccroides, Aster, Bromus*	18,138
19	Mamoura	40	80	30° 3.108	31° 17.518	2	Gravel, Loamy sand	Solid waste, between railway and car road, Fallow fields nearby Car road, Train road, Car park, Plant Nursing, Wastes and fire	Urbanized, Agroecosystem	≥78	*Malva, Avena, Bromus, Alhagi*	18,138
20	Toson	60	80	30° 3.612	31° 18.743	-1.5	Gravel, Loamy sand	Solid waste, nearby Military campus	Urbanization, Natural	≥78	*Avena, Echium, Bromus, Medicago*	18,138
21	Gheit El Enab	70	300	29° 54.755	31° 10.802	4	Clay, Loam with gravel	Nearby human settlements, Dumping waste, Grazing	Natural, Urbanization	50-75	*Alhagi, Aster, Chenopodium, Bromus, Avena*	22,663
22	Karmooz behind Bashayer El Khair	50	250	29° 54.018	31° 10.475	1	Clay, Loam with gravel	Heavy traffic, dumbing of solid waste	Natural, Pollution	50-70	*Alhagi, Cynodon, Atriplex, Brassica*	35,092
23	Karmooz at the edge of Bashayer El Khair	50	250	29° 53.675	31° 10.393	4	Clay, Loam with gravel	Heavy traffic of Cargo train, Dumbing of solid waste, fire, Multiline of railway, Construction activities	Natural, Urbanization	25-50	*Cressa, Avena, Alhagi*	35,092
24	El Kabbari	50	250	29° 53.281	31° 10.401	0	Clay	Dumping rubel material (cotton), Dumping sites	Natural, Urbanization		*Cynancum, Atriblex, Urtica uri, Lacuca*	35,092

Stand No.	Location	Stand size (m^2)		Coordinates		Alt. (m)	Soil Description	Human Impact	Type of Urbanization	Total Cover (%)	First Dominant Species	Population Density/ km^2 in 2019
		W	L	Long.	Lat.							
								for solid waste, infrastructure				
25	El Max	30	100	29° 51.673	31° 9.498	2	Compact soil (Loamy sandy)	Demolished waste, Organic waste	Industrial		*Emex, Imperata, Conyza, Mesembryanthemum*	26969
26	Al Wardiyan	20	80	29° 52.353	31° 10.076	1	Compact clayey loamy with pebbles	Organic waste, solid waste, domestic waste, demolished waste rubbish	infrastructure	5-25	*Conyza, Discroides, Malva, Lolum, Emex*	26969
27	El Kabbari (Protected)	20	100	29° 52.926	31° 10.572	11	Compact clayey Loamy	Traffic	Industrial +infrastructure	50-75	*Emex, Malva, Cynodon*	26969
28	El Kabbari	20	50	29° 55.248	31° 12.678	13	Compact clayey sandy	Traffic	Industrial	5-25	*Cynodon, Sismbrium, Lolium*	26969
29	Muharm bek-Al Nozha	30	40	29° 92.664	31° 199.388	12	Compact	Traffic, Human waste, organic waste, rubbish	Agroecosystem	50-75	*Hordium, Withania, Coniza, Malva*	22663
30	Al Qaaed Ibrahim Mosque & Faculty of Medicine	15	100	29° 54.223	31° 12.172	7	Compact soil with gravels clayey loamy	Settlements, Traffic pollution	infrastructure + traffic pollution	10-15	*Chenopodium, Cynodon*	21376
31	At Faculty of Commerce	15	100	29° 54.564	31° 12.322	6	Compact (Soil clay silty)	Settlements, Traffic pollution, Solid waste	infrastructure	10-15	*Hordium, Avena, Cynodon, Sismbryum*	21376
32	Shatibi to Shobana Muslimeen	15	300	29° 54.654	31° 12.370	10	Compact (clayey silty)	Settlements, Traffic pollution, Solid waste	infrastructure	25-30	*Urospermum, Chenopodium*	42289
33	Camp Cesar	15	200	29° 55.261	31° 12.692	19	Compact with pebbles and gravels	Low impact of solid waste	infrastructure	45-50	*Bromus uni, Hordeum, Urospermum, Chenopodium*	42289
34	El-Ibrahemia to Sporting	15	300	29° 55 23.595	31° 12 51.160	20	Compact, sand with dominant gravels		infrastructure	70-80	*Malva, Emex, Cynodon*	42289
35	El-Ibrahemia to Sporting Slope	15	300	29° 55 20.595	31° 12 51.160	17	Rocky man made		Man-made rocky slope	75	*Richardia*	42289
36	Moustafa Kamel	15	100	29° 56.970	31° 13.708	471			Wastes	50-75	*Malva, Urospermum*	15025
37	El-Ibrahemia to Sporting	30	150	29° 55 47.890	31° 13 81.874	20	Compact clay loamy	Solid waste, small markets	Type of agriculture practice of ornamental practice	50	*Cynodon, Urospermum, Hordium*	42289
38	Cleopatra to Sidi Gaber	20	100	29° 56 13.535	31° 13 15.171	10	Compact clay	Solid waste, settlements	Natural	60-70	*Hordeum, Malva, Urospermum, Avena*	15025
39	Moustafa Kamel	10	150	29° 56 53.476	31° 13 35.771	32	Compact Loamy clayey	Solid waste	Natural	30-40	*Chenopodium, Oryzopsis, Hordium*	15025

Stand No.	Location	Stand size (m^2)		Coordinates		Alt. (m)	Soil Description	Human Impact	Type of Urbanization	Total Cover (%)	First Dominant Species	Population Density/ km² in 2019
		W	L	Long.	Lat.							
40	Fleming-Bacos-Schutz	10	150	29° 96 29.610	31° 23 42.670	7	Compact with Pebbles clay loamy	Solid waste, Infrastructure	Natural	15-20	*Chenopodium, Hordeum, Cynodon*	68115
41	Gleem-San Stefano	10	200	29° 57 31.807	31° 14 18.050	8	Compact clay sandy	Solid waste	Natural	50-75	*Hordeum, Melilotus*	68115
42	Louran-Victoria	10	300	29° 97 34.850	31° 25 10.360	8	Compact Clayey loamy sandy	Solid waste	Natural	25-50	*Chenopodium, Oryzopsis, Sonchus, Malva*	68115
43	Victoria	75	150	29° 58 48.089	31° 14 56.201	24	Clayey loamy	Traffic pollution, Market area, little solid waste	Natural	0.5- >1	*Cynodon, Chenopodium*	26052
44	El Soyof	75	200	29° 58 43.669	31° 14 56.644	96	Loamy sandy	Infrastructure, Solid waste, overpopulation size	Natural	5	*Oryzopsis, Chenopodium*	26052
45	Sidi Bisher	50	200	29° 58 33.339	31° 15 10.090	89	Compact Sandy loamy	Infrastructure, Market area, Solid waste, overpopulation size	Natural	3-5	*Conyza, Hordeum*	68115
46	El Saraya	70	250	29° 58 23.345	31° 15 1.686	36	Compact loamy sandy	Infrastructure, Solid waste	Natural	5-10	*Phoenix, Hordeum*	26052
47	Tharwat	50	150	29° 58 09.615	31° 14 50.805	12	Sandy loamy (Gravely)	Infrastructure, Markets	Natural	5-10	*Chenopodium, Tribulus, Hordeum*	68115
48	San Stefano	80	200	29° 58 20.650	31° 14 38.401	37	Loamy Silty sandy	Traffic pollution, Big Market area, Infrastructure	Natural	3-5	*Sismbrium, Cynodon*	68115
49	Shots	75	150	29° 58 17.016	31° 14 23.752	53	Sandy loamy with pebbles	Overpopulation size, Fish market, pollution, Infrastructure, Caffe	Natural	0.5-1	*Lactuca, Hordeum*	68115
50	Safr	40	100	29° 58 12.148	31° 14 15.683	25	Clayey loamy with sand	Solid waste, Sewage pollution	Natural	5-10	*Cynodon, Avena, Oryzopsis*	68115
51	Bakus	100	250	29° 58 03.554	31° 14 07.083	36	Clayey loamy with pebbles	Traffic pollution, Market area, solid organic waste, Over population	Natural	5	*Cynodon, Sismbrium, Hordoum*	68115

Table S2. Recorded plant species in the present study. Status is: Inv.: invasive, Cau.: causal and Nat.: naturalized. Local distribution is: N: Nile region, O: Oases, M: Mediterranean coast, D: Desert, R: Red Sea coast, G: Gebel Elba, S: South Sinai. Life forms are: PH: Phanerophytes, CH: Chamaephytes, GE: Geo-Helophytes, HC: Hemicryptophytes, PA: Parasites, TH: Therophytes. The chorotypes are: SA: Saharo-Arabian, SU: Sudano-Zambezian, IT: Irano-Turanian, ME: Mediterranean, TR: Tropical, COSM: Cosmopolitan, EU: European, PAN: Pantropic, PAL: Paleotropical. Vegetation groups (VG) I: *Urospermum picroides*, II: *Chenopodium murale*, III: *Malva parviflora*, IV: *Cynodon dactylon*, V: *Hordeum leporinum* and VI: *Sonchus oleraceus*.

Family	Species	Status	Local Dist.	Life form	Chorotype	Habitat			Vegetation groups					
						Train	Tram	Total	I	II	III	IV	V	VI
Fabaceae	*Acacia saligna* (Labill.) H.L. Wendl.	Alien,Inv		PH	PAN	4.2		2.0		8.3				
Poaceae	Aegilops bicornis (Forssk.) Jaub.& Sp.	Weed	N,M	TH	ME,SA	4.2		2.0						33.3
Poaceae	*Aegilops ktschyi* Boiss.	Weed	O,M,S	TH	SA+IT	29.2	33.3	31.4				93.3	9.1	33.3
Fabaceae	*Alhagi graecorum* Boiss.	Weed	N,O,M,D,R,S	H	ME+IT	37.5	14.8	25.5		25.0	100.0	20.0		100
Aloeaceae	*Aloe vera* (L.) Burm.f.	Alien,Cau		H	ME+SA	4.2		2.0	16.7					
Malvaceae	*Althaea ludwigii* L.	Alien,Cau	N,O,M,D,S	TH	SA+IT+SU	8.3	3.7	5.9		25.0				
Amaranthaceae	*Amaranthus hybridus* subsp. *hybridus* L.	Alien,Nat	N,O,M,S	TH	TR	20.8	40.7	31.4	33.3	25.0	25.0	20.0	63.6	
Amaranthaceae	*Amaranthus lividus* L.	Alien,Nat	N,M,S	TH	TR	25.0	14.8	19.6		16.7		40.0	9.1	33.3
Amaranthaceae	*Amaranthus retroflexus* L.	Alien,Nat	N,M,S	TH	COSM	4.2		2.0		8.3				
Amaranthaceae	*Amaranthus viridis* L.	Weed	N,O,M,D,S	TH	COSM	8.3	11.1	9.8		0.0		26.7	9.1	
Apiaceae	*Ammi majus* L.	Weed	N,O,M,S	TH	ME	8.3		3.9		8.3	25.0			
Primulaceae	*Anagallis arvensis* L.	Weed	N,O,M,D,R,GE,S	TH	ME+IT+EU	4.2		2.0						33.3
Brassicaceae	*Anastatica hierochuntica* L.	Natural	O,D,R,S	TH	SA	4.2		2.0		8.3				
Boraginaceae	*Anchusa milleri hispida* Forssk. Willd.	Natural	M,D,S	TH	SA + IT	8.3		3.9				13.3		
Chenopodiaceae	*Arthrocnemum macrostachyum* (Moric.) C, Koch	Natural	N,O,M,D,R,S	CH	ME + SA	12.5		5.9		8.3	25.0	6.7		
Poaceae	*Arundo donax* L.	Alien,Cau	N,O,M,D,S	PH	ME+IT	4.2		2.0						33.3
Fabaceae	*Astragalus hamosus* L.	Natural	M,D	TH	ME + IT	4.2		2.0		8.3				
Asteraceae	*Atractylis carduus* (Forssk.) C. Chr.	Weed	M,D,GE,S	CH	SA	4.2		2.0		8.3				
Chenopodiaceae	*Atriplex canescens* Forssk.James	Alien,Nat	M,D	CH	PAN	8.3		3.9						66.7
Chenopodiaceae	*Atriplex coriacea* Forssk.	Natural	M,D	CH	SA+ME+SU	8.3		3.9			50.0			
Chenopodiaceae	*Atriplex halimus* L.	Natural	M,D,S	CH	SA+IT	8.3		3.9						66.7
Chenopodiaceae	*Atriplex leucoclada* var. *inamoena* (Allen) Zohary	Weed	O,M,D,S	CH	SA+IT	8.3		3.9		8.3	25.0			
Chenopodiaceae	*Atriplex lindleyi* Moq. Subsp. *inflata* (F. Muell.) P.G. Wilson	Alien,Nat	N,M,D,S	CH	PAN	8.3		3.9			50.0			
Chenopodiaceae	*Atriplex suberecta* Verd.	Alien,Nat	N,M,D	TH	PAN	12.5		5.9			50.0			33.3
Poaceae	*Avena barbata* Pott ex Link	Weed	N,O,M,D,S	TH	ME	50.0	7.4	27.5		50.0	75.0	26.7		33.3
Poaceae	*Avena fatua* L.	Weed	N,O,M,D,S	TH	PAL	58.3	48.1	52.9	66.7	8.3	50.0	80.0	45.5	100.0
Chenopodiaceae	*Bassia indica* (Wight) A.J. Scott	Alien,Inv	N,O,M,D	TH	SU+ IT	16.7	3.7	9.8			50.0			100.0
Chenopodiaceae	*Bassia muricata* (L.) Asch. In Schweinf.	Weed	O,M,D,S	TH	SA+IT	16.7	3.7	9.8		25.0	25.0			33.3
Fabaceae	*Bauhinia variegata* (L.) Benth L.	Alien,Cau		PH	TR	4.2		2.0		8.3				
Chenopodiaceae	*Beta vulgaris* L.	Weed	N,O,M,D,S	TH	ME+IT+EU	8.3		3.9		8.3	25.0			
Bombacaceae	*Bombax ceiba* L.	Alien,Cau		PH	TR		3.7	2.0	16.7					
Nyctaginaceae	*Bougainvillea glabra* Choisy in DC.	Alien,Cau		PH	TR		3.7	2.0					9.1	

Family	Species	Status	Local Dist.	Life form	Chorotype	Habitat			Vegetation groups					
						Train	Tram	Total	I	II	III	IV	V	VI
Poaceae	*Brachypodium distachyum* (L.) P. Beauv.	Weed	N,O,M,De,GE,S	TH	ME+IT	4.2	7.4	5.9				6.7	18.2	
Brassicaceae	*Brassica nigra* (L.) Koch in Röhling	Weed	N,O,D	TH	EU+ME	8.3	3.7	5.9		25.0				
Poaceae	*Bromus catharticus* Vahl	Alien,Nat	N,O,M,De	TH	ME+IT+EU	8.3	7.4	7.8				26.7		
Poaceae	*Bromus fasciculatus* C. Presl.	Natural	M,Di,S	TH	ME	4.2		2.0		8.3				
Poaceae	*Bromus inermis* Leyss.	Alien,Nat	N	H	ME+IT	33.3	14.8	23.5		58.3	75.0	6.7		33.3
Poaceae	Bromus madritensis L.	Natural	M,Di,S	H	ME + IT	4.2		2.0		8.3				
Poaceae	*Bromus rubens* Jusl. ap. L.	Natural	M,O,Di,S.	TH	ME+IT+SA	41.7	14.8	27.5	33.3	16.7	75.0	26.7	18.2	33.3
Brassicaceae	*Carrichtera annua* (L.) DC.	Natural	N,M,D,S	TH	ME		3.7	2.0		8.3				
Casuarinaceae	*Casuarina equisetifolia* Forest.&Forest.f.	Alien,Cau		PH	PAL		3.7	2.0					9.1	
Poaceae	*Cenchrus ciliaris* L.	Alien,Cau	N,M,D,R,S	H	ME+SA + SU	4.2	3.7	3.9		8.3				33.3
Asteraceae	*Centaurea aegialophila* Wagenitz	Natural	M	H	ME	8.3		3.9		8.3	25.0			
Asteraceae	*Centaurea alexandrina* Del.	Natural	M	TH	ME+SA	4.2		2.0		8.3				
Asteraceae	*Centaurea ammocyanus* Boiss.	Natural	Di	TH	ME+SA+IT +SU	8.3		3.9						66.7
Asteraceae	*Centaurea calcitrapa* L.	Alien,Cau	N,O,M	TH	SA	20.8	3.7	11.8		33.3	25.0	6.7		
Asteraceae	*Centaurea dimorpha* Viv.	Natural	M	H	ME	4.2		2.0				6.7		
Asteraceae	*Centaurea glomerata* Vahl.	Natural	M,O,D	TH	ME	4.2		2.0	16.7					
Asteraceae	*Centaurea solstitialis* L.	Natural	M,S	TH	IT + SA	4.2		2.0		8.3				
Chenopodiaceae	*Chenopodium album* L.	Weed	N,O,M,D,S	TH	COSM	25.0	48.1	37.3		8.3		80.0	54.5	
Chenopodiaceae	*Chenopodium ambrisoides* L.	Alien,Nat	N,O,M,S	TH	COSM	12.5	18.5	15.7	16.7	8.3	25.0	13.3	27.3	
Chenopodiaceae	*Chenopodium ficifolium* Sm.	Weed	N	TH	ME+ EU+ IT+PAL	4.2		2.0			25.0			
Chenopodiaceae	*Chenopodium murale* L.	Weed	N,O,M,D,R,GE,S	TH	COSM	79.2	88.9	84.3	83.3	100.0	50.0	93.3	81.8	33.3
Chenopodiaceae	*Chenopodium opulifolium* Schrad. Ex Koch & Ziz.	Weed	N	TH	ME+IT+EU +PAL	4.2	3.7	3.9				6.7		33.3
Asteraceae	*Cichorium endivia* subsp. *divaricatum* (Schoush.) P.D. Sell.	Weed	N,O,M	TH	ME+IT	4.2	7.4	5.9		25.0				
Cucurbitaceae	*Citrullus colocynthis* (L.) Schrad.	Weed	N,O,M,D,R,GE,S	H	SA	8.3		3.9				13.3		
Rutaceae	*Citrus aurantium* L.	Alien,Cau		PH	PAN		3.7	2.0		8.3				
Convolvulaceae	*Convolvulus arvensis* L.	Weed	N,O,M,De,S	GH	COSM	25.0	7.4	15.7		41.7	50.0	6.7		
Asteraceae	*Conyza aegyptiaca* (L.) Ait.	Weed	N,O,GE	TH	SU	25.0	7.4	15.7		33.3	25.0	20.0		
Asteraceae	*Conyza bonariensis* (L.) Cronquist	Alien,Nat	N,O,M,D,S	TH	PAN	29.2	3.7	15.7	16.7		75.0	13.3		66.7
Apiaceae	*Coriandrum sativum* L.	Alien,Cau	N,O,M	TH	IT+ME	4.2	3.7	3.9	16.7	8.3				
Brassicaceae	*Coronopus didymus* (L.) Sm.	Alien,Nat	Nd	TH	PAN	4.2	7.4	5.9	16.7	8.3		6.7		
Brassicaceae	*Coronopus squamatus* (Forssk.) Asch.	Weed	N,O,M	TH	ME+EU+IT	4.2	3.7	3.9	16.7					33.3
Convolvulaceae	*Cressa cretica* L.	Natural	N,O,M,D,R,GE,S	H	ME + IT	4.2	7.4	5.9				13.3		33.3
Convolvulaceae	*Cuscuta palaestina* Boiss.	Weed	M,De,S	PA	ME+SA	8.3	3.7	5.9			25.0	6.7		33.3
Asclepiadaceae	*Cynanchum acutum* L.	Weed	N,O,M	PH	ME+IT	41.7	14.8	27.5		16.7	100.0	33.3		100.0
Poaceae	*Cynodon dactylon* (L.) Pers.	Weed	N,O,M,D,R,GE,S	GH	COSM	75.0	81.5	78.4	50.0	75.0	75.0	93.3	72.7	100.0
Cyperaceae	*Cyperus rotundus* L.	Weed	N,O,M,D,R,GE,S	GH	TR	4.2	3.7	3.9		16.7				
Fabaceae	*Dalbergia sisso* Roxb. ex DC.	Alien,Inv		PH	PAL		3.7	2.0	16.7					
Solanaceae	*Datura innoxia* Mill.	Alien,Nat	N	TH	COSM	12.5	7.4	9.8	16.7	25.0			9.1	
Brassicaceae	*Descurainia sophia* (L.) Webb ex Prantl	Natural	M	TH	IT + SA	4.2		2.0			25.0			
Poaceae	*Digitaria sanguinalis* (L.) Scop.	Weed	N,O,M,De,S	TH	COSM	16.7	7.4	11.8			25.0	20.0	18.2	

Family	Species	Status	Local Dist.	Life form	Chorotype	Habitat			Vegetation groups					
						Train	Tram	Total	I	II	III	IV	V	VI
Brassicaceae	*Diplotaxis harra* (Forssk.) Boiss.	Weed	O,D,S	CH	SA	33.3	7.4	19.6	66.7	33.3		13.3		
Apiaceae	*Duacus syrticus* Murb.	Natural	M	TH	ME	4.2	7.4	5.9		8.3			18.2	
Poaceae	*Echinochloa colona* (L.) Link	Weed	N,O,M,D,R,GE,S	TH	TR	8.3		3.9			25.0			33.3
Asteraceae	*Echinops spinossimus* Turra.	Natural	M,D,R,S	H	ME	4.2		2.0		8.3				
Boraginaceae	*Echium angustifolium* Mill. subsp. *sericeum* Vahl	Natural	M,D,S	CH	ME	8.3	3.7	5.9		16.7	25.0			
Polygonaceae	*Emex spinosa* (L.) Campd.	Weed	N,O,M,Di	TH	SA +ME	58.3	40.7	49.0	66.7	91.7	50.0	46.7		33.3
Brassicaceae	*Enarthrocarpus strangulatus* Boiss.	Natural	M,D,S	TH	SA	8.3	7.4	7.8		16.7		6.7	9.1	
Geraniaceae	*Erodium crassifolium* L'Hér.	Natural	M,D,S	H	ME+IT	20.8	25.9	23.5				60.0	18.2	33.3
Geraniaceae	*Erodium glaucophyllum* (L.) L'Hér. In Aiton	Natural	Nv,M,D,S	H	IT + SA	12.5	22.2	17.6				13.3	54.5	33.3
Geraniaceae	*Erodium laciniatum* subsp. *pulverlentum* (Boiss.) Batt. & Trab.	Weed	N,M,D,R,S	TH	ME	20.8	14.8	17.6	16.7	33.3	25.0	13.3	9.1	
Geraniaceae	*Erodium oxyrhynchum* M. Bieb.	Natural	N,O,M,D,S	TH	SA	25.0	11.1	17.6	16.7	50.0			18.2	
Brassicaceae	*Eruca sativa* Mill.	Alien,Nat	N,O,M,D,S	TH	ME+IT	29.2	14.8	21.6		58.3		26.7		
Brassicaceae	*Erucaria pinnata* (Viv.) Täckh. & Boulos	Weed	O,M,D	TH	SA+ME		7.4	3.9				6.7	9.1	
Fabaceae	*Erythrina corallodendrum* L.	Alien,Cau		PH	PAN	4.2	3.7	3.9	16.7	8.3				
Myritaceae	*Eucalyptus camaldulensis* Dehn.	Alien,Cau		PH	PAN	8.3	7.4	7.8		8.3		6.7	18.2	
Euphorbiaceae	*Euphorbia arguta* Banks & Sol.	Weed	N,O,Dl,M	TH	EU+ME	12.5		5.9		25.0				
Euphorbiaceae	*Euphorbia heterophylla* L.	Alien,Nat	N	TH	PAN	4.2		2.0				6.7		
Euphorbiaceae	*Euphorbia peplus* L.	Weed	N,O,M,D,S	TH	ME+IT+EU	33.3	44.4	39.2	66.7	50.0		20.0	63.6	
Euphorbiaceae	*Euphorbia prostrata* Aiton	Alien,Nat	N,M,S	TH	PAN	4.2		2.0		8.3				
Zygophyllaceae	*Fagonia cretica* L.	Natural	M	CH	ME		3.7	2.0				6.7		
Zygophyllaceae	*Fagonia indica* Burm.f.	Natural	O,Da,R,GE	CH	SA	4.2		2.0		8.3				
Moraceae	*Ficus carica* L.	Alien,Nat	S	PH	ME+IT	4.2	3.7	3.9		8.3				33.3
Moraceae	*Ficus cycomorus* L.	Natural	N,O,M,S	PH	ME	4.2	7.4	5.9		8.3			18.2	
Moraceae	*Ficus microcarpa* L.*f.* cv. 'nitida'	Alien,Cau		PH	PAN	29.2	25.9	27.5	83.3	8.3		13.3	45.5	33.3
Moraceae	*Ficus palmata* Forssk.	Natural	De,GE,S	PH	SU	4.2		2.0		8.3				
Asteraceae	*Filago mareotica* Del.	Natural	M	TH	ME	4.2		2.0				6.7		
Fumariaceae	*Fumaria densiflora* DC.	Weed	N,O,M,Di	TH	ME+EU+IT	4.2	3.7	3.9				13.3		
Fumariaceae	*Fumaria judaica* Boiss.	Weed	Nd,M	TH	ME	16.7		7.8		33.3				
Rubiaceae	*Galium tricornutum* Dandy	Weed	Nd,O,M,Di,S	TH	ME	8.3		3.9		8.3				33.3
Asteraceae	*Glebionis coronaria* (L.) Tzvelev	Natural	N,M,S	TH	ME	8.3	3.7	5.9		8.3	25.0	6.7		
Caryophyllaceae	*Herniaria hemistemon* J. Gay	Natural	M,Di,S	H	ME + SA		7.4	3.9		8.3		6.7		
Malvaceae	*Hibiscus rosa-sinensis* L.	Alien,Cau		PH	PAN	4.2	7.4	5.9				13.3		33.3
Poaceae	*Hordeum leporinum* Link	Weed	N,O,M,Di,S	TH	IT+ ME	29.2	70.4	51.0	16.7		25.0	73.3	100.0	66.7
Poaceae	*Hordeum murinum* L. subsp. *galucum* (Steud.) Tzvelev	Weed	N,O,M,D,S	TH	ME+IT	58.3	33.3	45.1	66.7	83.3	50.0	33.3	9.1	33.3
Poaceae	*Hordeum vulgare* L.	Alien,Cau	N,M,D,S	TH	ME	12.5	3.7	7.8	50.0	8.3				
Solanaceae	*Hyoscyamus albus* L.	Natural	M,Da	H	ME+PAN		18.5	9.8				13.3	27.3	
Solanaceae	*Hyoscyamus muticus* L.	Natural	N,O,M,D,R,GE,S	CH	SA	4.2		2.0				6.7		
Arecaceae	*Hyphaene thebaica* (L.) Mart.	Natural	N,O,D,R,S	PH	SU + SA		3.7	2.0		8.3				
Poaceae	*Imperata cylindrica* (L.) Raeusch.	Weed	N,O,M,D,R,S	H	ME+SA+IT	8.3	3.7	5.9		8.3	25.0			33.3
Convolvulaceae	*Ipomoea caireca* (L.) Sweet	Alien,Cau	N,M,D	H	TR	12.5		5.9		16.7				33.3

Family	Species	Status	Local Dist.	Life form	Chorotype	Habitat			Vegetation groups					
						Train	Tram	Total	I	II	III	IV	V	VI
Convolvulaceae	*Ipomoea carnea* Jacq.	Alien,Inv	N,M	CH	PAN	4.2		2.0				6.7		
Juncaceae	*Juncus acutus* L.	Weed	N,O,M,De,S	H	ME+IT		3.7	2.0			25.0			
Juncaceae	*Juncus rigidus* Desf.	Weed	N,O,M,D,R,GE,S	H	SA+IT		3.7	2.0			25.0			
Asteraceae	*Lactuca serriola* L.	Weed	N,O,S	TH	ME+IT+EU	58.3	59.3	58.8	50.0	33.3	100.0	73.3	54.5	66.7
Verbinaceae	*Lantana camara* L.	Alien,Nat	N	PH	TR	8.3		3.9		16.7				
Asteraceae	*Launaea fragilis*	Natural	N,M,D	H	ME + SA	4.2		2.0						33.3
Asteraceae	*Launaea nudicaulis* (L.) Hook. F.	Weed	N,O,M,D,R,GE,S	H	SA	8.3		3.9						66.7
Asteraceae	*Launaea resedifolia* (L.)Kuntze	Natural	M	H	ME	4.2		2.0				6.7		
Linaceae	*Linum strictum* L.	Weed	M	TH	ME+EU+SU+IT		3.7	2.0		8.3				
Brassicaceae	*Lobularia arabica* (Boiss.) Muschl.	Weed	M,D	TH	SA	4.2		2.0			25.0			
Brassicaceae	*Lobularia libyca* (Viv.) Meissn.	Weed	M,D	TH	ME	12.5		5.9		25.0				
Poaceae	*Lolium perenne* L.	Weed	N,O,M,Di,S	H	EU+ME+IT	16.7	18.5	17.6	33.3	16.7	25.0	13.3	9.1	33.3
Poaceae	*Lolium temulentum* L.	Weed	Nd,O,M,Di,S	TH	EU+ME+IT	8.3		3.9						66.7
Solanaceae	*Lycoperscon esculentum*	Alien,Cau		H	COSM	33.3	29.6	31.4	83.3	16.7		33.3	27.3	33.3
Malvaceae	*Malva aegyptia* L.	Weed	M,Mp,Di	TH	SA+ME	12.5		5.9			25.0			66.7
Malvaceae	*Malva parviflora* L.	Weed	N,O,M,D,R,S	TH	ME+IT	79.2	74.1	76.5	33.3	100.0	100.0	80.0	72.7	33.3
Malvaceae	*Malva sylvestris* L.	Weed	Nd,M,Di	H	EU+ME+IT	8.3	22.2	15.7		8.3	25.0	26.7	18.2	
Fabaceae	*Medicago laciniata* (L.) Mill.	Weed	M,Di,S	TH	SA	4.2		2.0		8.3				
Fabaceae	*Medicago polymorpha* L.	Weed	M,O,Di	TH	ME+IT+EU	16.7		7.8		33.3				
Fabaceae	*Melilotus alba* Medic.	Weed	Nd,Mp	Bi	EU+IT+ME	4.2		2.0		8.3				
Fabaceae	*Melilotus indicus* (L.) All.	Weed	N,O,M,D,S	TH	ME	25.0	18.5	21.6	16.7	50.0	25.0	13.3	9.1	
Lamiaceae	*Mentha longifolia* (L.) Hudds.	Weed	N,O,S	H	ME+IT+EU		3.7	2.0				6.7		
Aizoaceae	*Mesembryanthemum crystallinum* L.	Natural	M,N,D	TH	ME+EU	20.8		9.8		25.0		6.7		33.3
Aizoaceae	*Mesembryanthemum nodiflorum* L.	Natural	M,N	TH	ME + EU+SA		3.7	2.0			25.0			
Fabaceae	*Mimosa pigra* L. Juslenius	Alien,Cau	Nv,Da	PH	SU	8.3		3.9				13.3		
Myritaceae	*Myrtus communis* L.	Alien,Cau		PH	IT	4.2		2.0		8.3				
Apocynaceae	*Nerium oleander* L.	Alien,Cau		CH	ME	4.2	11.1	7.8	16.7	8.3		13.3		
Solanaceae	*Nicotiana glauca* Graham	Alien,Nat	N,O,M,De,S	PH	COSM	41.7	22.2	31.4	16.7	33.3	50.0	20.0	36.4	66.7
Lamiaceae	*Ocimum basilicum* L.	Alien,Cau		TH	TR		3.7	2.0		8.3				
Oleaceae	*Olea europaea* L. var. *europaea*	Natural	M,D	PH	SA+ME	4.2	3.7	3.9	16.7	8.3				
Resedaceae	*Oligomeris linifolia* (Vahl) Macbr.	Weed	N,M,D,R,S	TH	SU	4.2		2.0			25.0			
Fabaceae	*Onobrychis crista-galli* (L.) Lam.	Natural	M,Di	TH	ME+SU	4.2		2.0				6.7		
Asteraceae	*Onopordum alexandrinum* Boiss.	Natural	M,S	H	ME	4.2		2.0		8.3				
Orobanchaceae	*Orobanche cernua* Loefl.	Natural	N,M,D,R,S	PA	ME+SA+IT	4.2		2.0						33.3
Poaceae	*Oryzopsis miliacea* (L.) Asch. & Schweinf.	Weed	N,M,De,S	CH	ME	29.2	55.6	43.1	16.7	8.3	25.0	80.0	54.5	33.3
Poaceae	*Panicum repens* L.	Weed	N,O,M,De	CH	TR		3.7	2.0		8.3				
Poaceae	*Parapholis marginata* Runemark	Natural	M,N,O,Di,S	TH	ME+IT+EU	4.2	3.7	3.9			25.0	6.7		
Urticaceae	*Parietaria alsinifolia* Delile	Natural	De,S	TH	SA	16.7	11.1	13.7	33.3	8.3		26.7		
Caryophyllaceae	*Paronychia arabica* (L.) DC.	Natural	O,M	TH	SA+ ME+SU	4.2		2.0		8.3				
Poaceae	*Pennisetum glaucum* (L.) R.Br.	Weed	N,O,De,GE	TH	TR		3.7	2.0		8.3				
Euphorbiaceae	*Pergularia tomentosa* L	Natural	GE,S	CH	SU	4.2		2.0				6.7		
Polygonaceae	*Perisicaria salicifolia*	Natural	N,M,R,S	GH	PAL	8.3	25.9	17.6		8.3	25.0	20.0	27.3	33.3

Family	Species	Status	Local Dist.	Life form	Chorotype	Habitat			Vegetation groups					
						Train	Tram	Total	I	II	III	IV	V	VI
Poaceae	*Phalaris minor* Retz.	Weed	N,O,M,S,D	TH	ME+IT	37.5	48.1	43.1	50.0	8.3	50.0	60.0	45.5	66.7
Poaceae	*Phalaris paradoxa* L.	Weed	N,M,Di	TH	ME+IT	4.2		2.0	16.7					
Palmae Arecaceae	*Phoenix dactylifera* L.	Natural	N,O,M,D,R,GE,S	PH	SA	37.5	77.8	58.8	50.0	33.3	25.0	66.7	90.9	66.7
Poaceae	*Phragmites australis* (Cav.) Trin. Ex Steud.	Weed	N,O,M,D,R,S	H	COSM	33.3	18.5	25.5		33.3	75.0	20.0	9.1	66.7
Verbinaceae	*Phyla nodiflora* (L.) Greene	Weed	N,O,M,D,S	H	ME+EU+TR	4.2		2.0		8.3				
Solanaceae	*Physalis ixocarpa* Brot. ex Hornem.	Alien,Nat	N	TH	PAN	8.3	3.7	5.9	16.7	16.7				
Asteraceae	*Picris asplenioides*	Weed	N,M,D,S	TH	SA	4.2		2.0						33.3
Plantaginaceae	*Plantago lagopus* L.	Weed	N,O,M	TH	ME+IT		3.7	2.0				6.7		
Plantaginaceae	*Plantago lanceolata* L.	Natural	Nd	H	COSM		3.7	2.0		8.3				
Plantaginaceae	*Plantago major* L.	Weed	N,O,M,S	TH	ME+IT+EU	4.2		2.0		8.3				
Asteraceae	*Pluchea dioscoridis* (L.) DC.	Weed	N,O,M,De,S	PH	SA+SU	37.5	51.9	45.1	66.7	8.3	100.0	40.0	54.5	66.7
Poaceae	*Poa annua* L.	Weed	N,O,M,S	TH	ME+IT+EU	12.5	14.8	13.7		8.3		26.7	18.2	
Poaceae	*Poa infirma* Kunth in Humb.	Weed	Nd,M,Da	TH	ME		3.7	2.0		8.3				
Fabaceae	*Poinciana rigia*	Alien,Cau		PH	SU+ME+PAN	8.3	14.8	11.8	16.7			13.3	27.3	
Polygonaceae	*Polygonum equisetiforme* Sm.	Weed	N,O,M,D,S	H	ME+IT	16.7	11.1	13.7	16.7	8.3	25.0	20.0	9.1	
Poaceae	*Polypogon monspeliensis* (L.) Desf.	Weed	N,O,M,D,R,S	TH	ME+SA+IT	8.3	11.1	9.8	33.3	16.7		6.7		
Portulacaceae	*Portulaca oleracea* L.	Weed	N,O,M,S	TH	COSM	4.2		2.0		8.3				
Fabaceae	*Prosopis juliflora* (Sw.) DC.	Alien,Inv		PH	SA	4.2		2.0		8.3				
Myritaceae	*Psidium guajava* L.	Alien,Cau		PH	TR		3.7	2.0		8.3				
Brassicaceae	*Raphanus raphanistrum* L.	Weed	N,M	TH	ME+EU		3.7	2.0		8.3				
Asteraceae	*Reichardia tingitana* (L.) Roth	Weed	N,M,D,R,GE,S	TH	ME+IT	66.7	29.6	47.1	66.7	83.3	50.0	46.7		33.3
Resedaceae	*Reseda alba* L.	Natural	Nv,M,Di,S	TH	ME	4.2		2.0						33.3
Euphorbiaceae	*Ricinus communis* L.	Alien,Nat	D,GE	PH	TR	37.5	7.4	21.6		25.0	50.0	20.0	9.1	66.7
Poaceae	*Rostraria cristata* (L.) Tzvelev	Weed	N,O,M,D,S	TH	ME + IT	20.8	11.1	15.7			25.0	26.7	9.1	66.7
Polygonaceae	*Rumex dentatus* L.	Weed	N,M	TH	ME+IT+EU	16.7	7.4	11.8	16.7	8.3		6.7	9.1	66.7
Polygonaceae	*Rumex pictus* Forssk.	Weed	M,Di	TH	SA + ME	4.2	7.4	5.9				6.7	9.1	33.3
Polygonaceae	*Rumex vesicarius* L.	Weed	M,D,R,GE,S	TH	SA	4.2	3.7	3.9				6.7		33.3
Chenopodiaceae	*Sarcocornia fruticosa* (L.) A.J. Scott	Natural	N,O,M,D	CH	ME + EU	4.2		2.0						33.3
Anacardiaceae	*Schinus mollis* L.	Alien,Nat		PH	PAN	8.3	11.1	9.8				13.3	27.3	
Poaceae	*Schismus barbatus* (L.) Thell	Weed	Nd,O,M,D,R,S	TH	ME + SA + IT	12.5	3.7	7.8		25.0		6.7		
Asteraceae	*Senecio aegyptius* L.	Weed	N,O,D	TH	ME+EU	4.2		2.0				6.7		
Asteraceae	*Senecio desfontainei* Druce	Weed	N,O,M,D,R,S	TH	SA+ME	66.7	51.9	58.8	66.7	75.0	50.0	66.7	27.3	66.7
Asteraceae	*Senecio flavus* (Decne) Sch. Bip.	Weed	D,R,GE,S	TH	ME+SA+SU		3.7	2.0		8.3				
Asteraceae	*Senecio glaucus* L.	Weed	N,O,M,D,R,S	TH	SA + IT	29.2	7.4	17.6		25.0		33.3		33.3
Asteraceae	*Senecio vulgaris* L.	Weed	N,O,M,D	TH	EU+IT+ME	4.2	14.8	9.8			25.0	13.3	18.2	
Fabaceae	*Senna italica* Mill.	Natural	N,O,D,GE,S	CH	SU	8.3	3.7	5.9		8.3		6.7	9.1	
Poaceae	*Setaria viridis* (L.) P. Beauv.	Alien,Nat	N,O,De,S	TH	ME+IT+EU	16.7	22.2	19.6	16.7		50.0		45.5	66.7
Caryophyllaceae	*Silene villosa* var. *villosa* Forssk.	Weed	N,M,S	TH	SA+IT	4.2		2.0		8.3				
Asteraceae	*Silybum marianum* (L.) Gaertn.	Weed	N,O,M,D	H	IT+EU+ME	4.2		2.0			25.0			
Brassicaceae	*Sinapis alba* L.	Weed	Nd,M	TH	ME+IT+EU	12.5		5.9	16.7		25.0			33.3

Family	Species	Status	Local Dist.	Life form	Chorotype	Habitat			Vegetation groups					
						Train	Tram	Total	I	II	III	IV	V	VI
Brassicaceae	*Sinapis allionii* Jacq.	Weed	N,M	TH	Endemic	4.2		2.0			25.0			
Brassicaceae	*Sinapis arvensis L. var. orientalis*	Weed	N,O,M,D,S	TH	ME	20.8	3.7	11.8		50.0				
Brassicaceae	*Sisymbrium erysimoides* Desf.	Weed	D,R,GE,S	TH	SA+ME+TR	8.3	3.7	5.9		16.7				33.3
Brassicaceae	*Sisymbrium irio* L.	Weed	N,M,De,GE,S	TH	ME+IT	54.2	74.1	64.7	83.3	75.0		80.0	54.5	33.3
Solanaceae	*Solanum nigrum* L.	Weed	N,O,M,D,R,GE,S	H	ME+IT+EU	12.5	7.4	9.8	33.3	8.3		6.7		33.3
Asteraceae	*Sonchus asper* (L.) Hill.	Weed	N,O,M,S	TH	ME	4.2	3.7	3.9		8.3		6.7		
Asteraceae	*Sonchus macrocarpus* Boulos et Jeffrey	Weed	Nd,M	CH	Endemic	8.3	3.7	5.9		25.0				
Asteraceae	*Sonchus oleraceus* L.	Weed	N,O,M,D,R,S	TH	ME+IT+EU	62.5	48.1	54.9	33.3	75.0		66.7	36.4	100.0
Caryophyllaceae	*Spergularia diandra* (Guss.) Boiss.	Weed	N,O,M,D,R,GE,S	TH	ME+SA+IT	4.2	11.1	7.8		25.0		6.7		
Caryophyllaceae	*Spergularia marina* (L.) Griseb.	Weed	N,M	H	EU+ME+IT	8.3	7.4	7.8	33.3			6.7		33.3
Poaceae	*Sprobolus spicatus* (Vahl) Kunth	Weed	N,O,M,D,R,S	GH	PAL	4.2	3.7	3.9		16.7				
Chenopodiaceae	*Suaeda pruinosa* Lange	Natural	M,Di	CH	ME		7.4	3.9		8.3	25.0			
Asteraceae	*Symphyotrichum squamatum* (Spreng.) Nesom	Alien,Nat	N,O,M,D,S	BI	PAN	20.8	3.7	11.8			75.0			100.0
Bignoniaceae	*Techoma stans* (L.) HBK	Alien,Cau		PH	PAN	4.2		2.0		8.3				
Antalaceae	*Thesium humile* var. *maritima* Simps.(N.D. Simpson) Sa'ad	Natural	N,O,D	TH	EU+ME	4.2		2.0						33.3
Zygophyllaceae	*Tribulus terrestris* L.	Weed	N,M,D,R,S	GH	ME+IT+EU	8.3	25.9	17.6	16.7			20.0	36.4	33.3
Boraginaceae	*Trichodesma africanum* (L.) R. Br.	Natural	N,O,M,D,R,GE,S	TH	SA	16.7		7.8		33.3				
Poaceae	*Triticum aestivum* L.	Alien,Cau	N	TH	EU+IT	12.5	3.7	7.8	16.7	8.3	25.0			33.3
Typhaceae	*Typha domingensis* (Pers.) Poir. Ex Steud.	Weed	N,O,M,D,R,S	GH	ME+SA+IT	4.2		2.0		8.3				
Asteraceae	*Urospermum picroides* (L.) F. W. Schmidt	Weed	N,O,M,D,GE,S	TH	ME+IT	70.8	70.4	70.6	83.3	66.7	50.0	86.7	54.5	66.7
Urticaceae	*Urtica pilulifera* L.	Weed	M,Nd	TH	ME + IT + EU	29.2	11.1	19.6	16.7	16.7	50.0	20.0		66.7
Urticaceae	*Urtica urens* L.	Weed	N,M,De	TH	ME+EU	29.2	11.1	19.6	33.3	16.7		33.3		33.3
Scrophulariaceae	*Veronica anagallis-aquatica* L.	Weed	N	H	COSM	20.8		9.8		16.7		20.0		
Fabaceae	*Vicia sativa* L.	Weed	N,O,M,Di,S	TH	ME+IT+EU	4.2		2.0		8.3				
Verbenaceae	*Vitex angus-castus* L.	Alien,Cau		PH	ME	8.3		3.9		16.7				
Vitaceae	*Vitis venifera* L.	Alien,Cau		PH	ME+EU	4.2		2.0						33.3
Arecaceae	*Washingtonia robusta* H. A. Wendl.	Alien,Cau		PH	ME+PAN	4.2	7.4	5.9			25.0		18.2	
Solanaceae	*Withania obtusifolia* Tackh.	Alien,Nat	GE,S	PH	SU	20.8	7.4	13.7	33.3	16.7		13.3		33.3
Poaceae	*Zea mays*	Alien,Cau	N	TH	PAL		3.7	2.0					9.1	
Rhamnaceae	*Ziziphus spina-christi* (L.) Desf.	Alien,Nat	N,O,M,D,R,GE,S	PH	SU	12.5	7.4	9.8		33.3	25.0			
						**197**	**139**	**224**	**58**	**142**	**72**	**104**	**60**	**81**
